# Extraction and Purification of the FrHb1 Fraction from Commercial Natural Latex of *Hevea brasiliensis* for Biomedical Applications

**DOI:** 10.3390/biomimetics10020085

**Published:** 2025-01-30

**Authors:** Ana Karoline Almeida da Silva, Gustavo Adolfo Marcelino de Almeida Nunes, Rafael Mendes Faria, Ana Luiza Coutinho Favilla, Jéssica Dornelas, Marcos Augusto Mountinho Fonseca, Angie Daniela Ibarra Benavides, Lindemberg Barreto Mota da Costa, Mário Fabrício Fleury Rosa, Adson Ferreira da Rocha, Suelia de Siqueira Rodrigues Fleury Rosa

**Affiliations:** 1 Postgraduate Programme in Mechatronic Systems, Mechanical Engineering Department, Campus Darcy Ribeiro, University of Brasilia, Brasilia 70910-900, DF, Brazil; gusnunes@gmail.com (G.A.M.d.A.N.); marcos.fonseca2@gmail.com (M.A.M.F.); ing.adib3@gmail.com (A.D.I.B.); sdf73@cornell.edu (S.d.S.R.F.R.); 2 Federal Institute of Education, Science and Technology of Triângulo Mineiro, Paracatu 38603-402, MG, Brazil; rafaelmendes@iftm.edu.br; 3 National Service for Industrial Learning, Center for Technology in the Chemical and Textile Industry (SENAI-CETIQT), SENAI Institute of Innovation in Biosynthetics and Fibers, Process Development Engineering Coordination, Rio de Janeiro 20261-245, RJ, Brazil; alcfavilla@cetiqt.senai.br; 4 Nano Onco 3D, Rio de Janeiro 20000-000, RJ, Brazil; dornelas.jessica@nanoonco3d.com.br; 5 Postgraduate Programme in Biomedical Engineering, Faculty of Science and Engineering Technologies (FCTE), University of Brasilia, Gama 72444-240, DF, Brazil; bergbmota1515@gmail.com (L.B.M.d.C.); mariorosafleury@gmail.com (M.F.F.R.); adson@unb.br (A.F.d.R.); 6 Graduate Program on Electrical Engineering, Department of Electrical Engineering, University of Brasilia, Darcy Ribeiro 70910-900, DF, Brazil; 7 Meinig School of Biomedical Engineering, Master of Engineering (M.Eng.) Program, Cornell University, Ithaca, New York, NY 14853, USA

**Keywords:** biomedical applications, natural rubber latex, regenerative medicine, biomaterial, affinity chromatography

## Abstract

Biomaterials interact with biological systems, influencing their responses. Different types of polymers—both natural and synthetic—are widely used in biomedical engineering, among a plethora of healthcare applications, to promote tissue regeneration. The natural rubber latex extracted from *Hevea brasiliensis* is a biopolymer that whose biocompatibility makes it a valuable study object. Its great regenerative properties are largely associated with the fraction FrHB1, which has demonstrated angiogenic and wound-healing potential by inducing blood vessel formation, collagen synthesis, and fibroblast migration—crucial factors for tissue repair. This study aimed to develop scalable methods for extracting and purifying the F1 protein fraction from industrialized natural latex for biomedical applications. We tested two types of industrial latex, bi-centrifuged and pre-vulcanized latex as well as 60% centrifuged natural latex to determine the most effective composition used in subsequent extractions and fractionation steps. Then, we isolated FrHB1 from the pre-vulcanized latex using selective precipitation, ultrafiltration, and affinity chromatography. The yield of the first batch of this serum was 40.62% with protein concentration of 1.52 ± 0.06 mg/mL. The second batch had a yield of 49.74%; however, due to results lying outside the analytical curve, its protein concentration could not be calculated. The yield of the third batch was 57.19%, and its protein concentration was 1.8477 ± 0.033 mg/mL. This approach facilitates large-scale therapeutic applications utilizing a commercially viable and accessible resource. Moreover, these findings highlight industrialized natural latex as a sustainable source of bioactive molecules, contributing to advancements in regenerative medicine and tissue engineering.

## 1. Introduction

Biomaterials refer to a group of materials designed to interact with biological systems, influencing responses in human tissues or organs [1]. Natural and synthetic polymers have been extensively applied in biomedical and tissue engineering contexts, where they are essential for promoting tissue regeneration, replacing injured tissues, serving as components in implants, and supporting various other healthcare applications [2].

**Figure 1:**
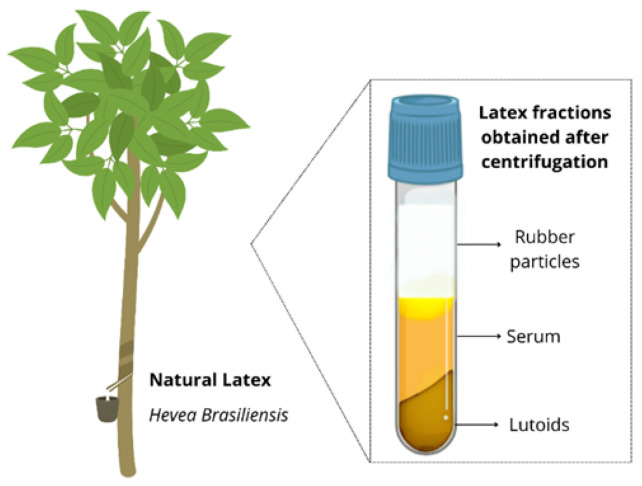
The natural latex from *Hevea brasiliensis* is present in vessels located in the inner cortex of the tree’s bark and is extracted through a process known as tapping. Centrifugation of the latex separates it into three fractions: the upper fraction, which contains rubber particles and influences its mechanical properties; the intermediate fraction, or C-serum, which is rich in proteins, salts, organic acids, nucleotides, and carbohydrates; and the lower fraction, or B-serum, which contains lutoids, calcium, magnesium, and Frey–Wyssling complexes (carotenoids and lipids) responsible for its yellowish coloration. Source: the authors, 2024.

Natural rubber latex (NRL) is a biopolymer extracted from *Hevea brasiliensis*, also known as the rubber tree. This tropical plant belongs to the Euphorbiaceae family and is native to the tropical forests of the Amazon basin. At present, it is the only commercial source of NRL. More than 60% of the dry NR production is used by the tire industry [3]. NRL is found in the form of a colloidal suspension, is manually extracted, and is primarily composed of natural rubber or isoprene (30–45%) and non-rubber fractions, including water (50%), proteins, lipids, carbohydrates, and inorganic salts [1,4] (Figure 1).

NRL has shown significant potential in medical applications, including controlled drug delivery systems, transdermal drug administration, and regenerative medicine, with a focus on the regeneration of skin, bones, cartilage, and other tissues [5]. Biocompatibility studies in animal models have demonstrated its effectiveness, as seen in subcutaneous implants in dogs, which resulted in the regeneration of host tissue without signs of fibrosis or rejection [6]. Additionally, NRL promoted effective tissue repair in dermal ulcers in rabbits, with results comparable to natural healing, and it has been successfully used in reconstructive surgeries, such as pericardium repair and the treatment of abdominal wall defects [7,8]. The biomaterial also induced the formation of new blood vessels in rabbit corneas, highlighting its angiogenic potential [9].

**Figure 2:**
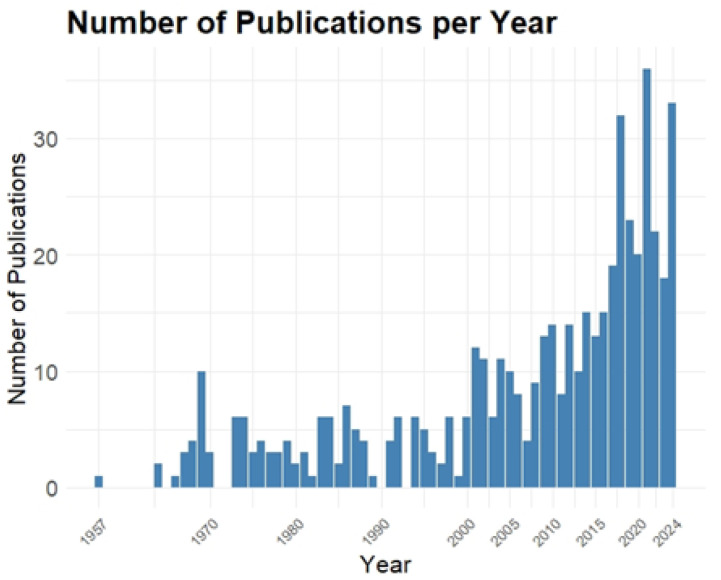
Bibliometric analysis showing the evolution of publications on the use of rubber in biomedicine, particularly in wound healing. The research, conducted on 20 October 2024, collected 225 results from PubMed, 359 from SCOPUS, and 151 from Embase, using keywords such as “Rubber”, “Hevea”, “Biomedical Research” and “Wound Healing”. A significant increase in publications was observed starting in 2017, with a peak in 2021, indicating a growing interest from the scientific community in the biomedical applications of rubber. Source: the authors, 2024.

A study evaluated the effect of a natural latex membrane on wound healing in the hard palate after free gingival graft removal. A total of 24 patients participated, and they were divided into two groups: one group received an acrylic plate with surgical cement, while the other received a plate with the latex membrane. Both groups showed a reduction in wound area, with the group treated with the latex membrane reporting a significant decrease in pain without additional effects on healing [10]. Another study investigated the Rapha^®^ system, which combines red light therapy and a natural latex biomembrane to treat neuropathic ulcers associated with diabetic foot problems. In a randomized clinical trial with 15 participants, the group treated with the Rapha^®^ system achieved higher average healing rates when compared to the standard protocol, demonstrating the system’s efficacy, even when applied by the patients themselves at home [11]. Additionally, we conducted a bibliometric analysis (Figure 2) on the index of publications on NRL in the field of biomedical engineering over the years, highlighting associated trends and advancements in research.

The serum proteins present in natural rubber latex (NRL) are divided into three main fractions: FrHB1 protein (or F1), FrHB2 protein (or F2), and FrHB3 protein (or F3). These fractions have the ability to interact directly with cells and living tissues, playing a crucial role in tissue regeneration. Specifically, the F1 protein fraction stands out due to its angiogenic effects, meaning its ability to promote the formation of new blood vessels [12]. Mendonça observed that the F1 shows greater activity of increasing vascular permeability when compared to the FrHB2 and FrHB3 fractions. Both also demonstrated the ability to increase vascular permeability, although not significantly [13]. This property is critical for the regeneration of damaged tissues, as increased vascularization facilitates the supply of nutrients and oxygen to recovering cells, accelerating the healing process. Furthermore, F1 stimulates cell growth, making it a promising tool for therapeutic applications focused on tissue regeneration, such as those for the treatment of nerve injuries and chronic wounds [14].

Morais et al. (2024) [14] compared the wound-healing potential of the F1 protein and natural rubber latex (NRL) serum in an in vivo model. The results indicated that both the F1 protein and NRL serum significantly stimulated fibroblast proliferation, with a concentration of 0.01% of the F1 protein showing the most pronounced effect. Histopathological analysis revealed that both treatments accelerated healing and tissue repair, with the F1 protein being particularly effective in stimulating collagen synthesis by fibroblastic cells. Although neither the F1 protein nor the NRL serum significantly altered inflammatory cytokine levels, the topical application of the F1 protein drastically reduced nitric oxide levels. These findings highlight the potential of both the F1 protein and the NRL serum as wound-healing promoters.

Additional studies support the wound-healing and regenerative effects of natural latex from *Hevea brasiliensis* and its F1 protein fraction. One study showed that 1% latex serum promoted more efficient re-epithelialization and thinner scabs in skin wounds, as well as stimulating cell migration and proliferation [15]. Another work investigated the F1 fraction in biomaterials for bone repair, observing that a concentration of 0.025% resulted in the greatest bone volume gain [16]. In sciatic nerve injuries, F1 demonstrated a regenerative effect superior to that of isolated laser therapy, standing out for significant motor recovery [17].

To the best of our knowledge, no prior studies have described a method for extracting and purifying the FrHb1 protein fraction from industrialized natural latex. This makes our study not only innovative but also a significant step toward optimizing latex-based biotechnological applications. Current methodologies rely exclusively on fresh latex directly harvested from trees, limiting scalability and standardization. By adapting existing purification techniques to industrialized latex, we introduce a new avenue for utilizing processed latex as a viable raw material. This approach facilitates large-scale production, reduces variability in raw material composition, and enhances translational research, bridging scientific discovery to commercial applications. Furthermore, by leveraging an industrially available source, we anticipate reductions in production costs, improvements in medical device manufacturing efficiency, and advancements in sustainable regenerative therapies.

## 2. Materials and Methods

A few hours after collection, natural latex undergoes a spontaneous coagulation process, where coagulated and aqueous phases form at the top and bottom, respectively. This phenomenon occurs due to the release of anions from fatty acids that adsorb onto the surface of rubber particles, interacting with divalent metal cations present in the latex, which favors coagulation. It involves reactions between cations and proteins, as well as the action of enzymes and micro-organisms that interact with non-rubber compounds, reducing their stabilizing capacity [4].

The choice of ammonia concentrations for latex stabilization is based on its essential role in maintaining colloidal stability and preventing microbial degradation. High-ammonia (HA) latex, containing between 0.6% and 0.7% ammonia, offers both stabilization and microbial inhibition. Low-ammonia latex (LA), with a concentration of ≤0.29%, requires additional preservatives, such as TMTD and ZnO, to compensate for the lower amount of ammonia [18].

Due to its rapid degradation and tendency to coagulate, natural latex is often stabilized through the addition of chemical agents. The most common preservative used with freshly collected latex for extended periods is ammonia. When added in concentrations above 0.35% by weight, ammonia acts as an effective bactericide, while lower concentrations (0.005% by weight) may promote bacterial growth. The latex serum was obtained using a procedure based on literature [19]. The latex (natural and pre-vulcanized) was diluted in distilled water at a 1:1 ratio and kept under stirring at room temperature (approximately 25 °C) for exactly 10 min using a magnetic stirrer (model C-MAG HS 7; IKA, China) for homogenization [4]. NRL has a molecular weight ranging from 10^4^ to 10^5^ kDa and contains a mixture of organic substances, including many different proteins that account for approximately 1% to 1.5% of its volume [1].

**Figure 3:**
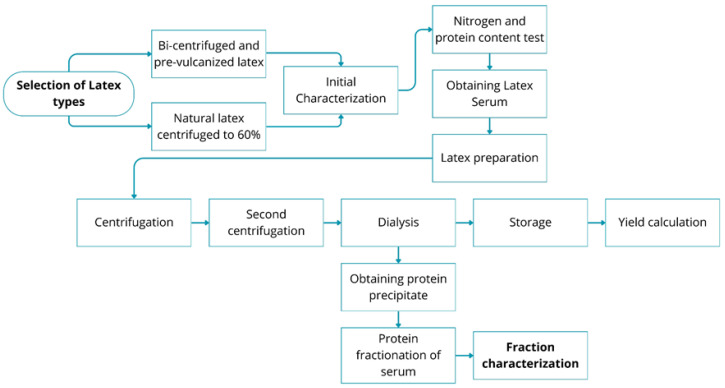
A flowchart of the methodology for extraction and characterization of the F1 protein fraction from latex, including selection of latex, initial tests, serum extraction, double centrifugation, dialysis for protein precipitation, storage, yield calculation, fractionation, and final characterization. Source: the authors, 2024.

Considering the characteristics of natural latex, we also decided to investigate the properties of industrialized latex, seeking to identify alternatives that could offer better performance in our analyses. For this purpose, we tested two types of industrial latex—bi-centrifuged and pre-vulcanized latex and 60% centrifuged natural latex—to determine the more suitable for the subsequent analyses in the project. The samples were characterized for nitrogen content and proteins, using precise methods such as the elemental analyzer and the Kjeldahl system. Figure 3 illustrates the methodology used.

The main objective of these tests was to ensure that the selected latex had the ideal chemical composition, especially concerning proteins, so that it could be effectively used in the subsequent extraction and fractionation steps. The comparison between the two types of latex allowed for identification of the most suitable one for the proposed procedures, ensuring greater precision and reproducibility in future experiments. The analyses were conducted with 4 L bi-centrifuged and pre-vulcanized latex samples and 2 L of 60% centrifuged natural latex from the Du Látex^®^ brand.

### 2.1. Characterization of the Sample by Determination of Nitrogen and Proteins

The determination of nitrogen was performed using the FlashSmart CHN/O elemental analyzer (Thermo Fisher Scientific Inc., Waltham, MA, USA) with the conventional operating methodology of the equipment and the BBOT standard $C_{26}H_{26}N_{2}SO_{2}$ (Thermo Fisher Scientific, Bremen, HH, Germany). The latex samples were prepared by drying in an oven at 50 °C for 120 min.

Organic nitrogen was also determined using the Kjeldahl digestion and distillation system (VELP Scientifica, Usmate Velate, MB, Italy). The method is a standard one and consists of three steps: digestion, distillation, and titration (ISO 1656, 2019) [20]. For the digestion step, KjTabs™ VCM tablets (3.5 g $K_{2}SO_{4}$ + 0.1 g $CuSO_{4}$· 5 $H_{2}O$), $H_{2}SO_{4}$, and 30% $H_{2}O_{2}$ were used. The following heating ramp was employed during this step: 30 min at 250 °C, 30 min at 350 °C, and 120 min at 420 °C. In the distillation step, 32% NaOH and 4% $H_{3}BO_{3}$ were used. Titration was performed with 0.2 N $H_{2}SO_{4}$ (standardized). In the calculation, a protein conversion factor of 6.25 (ISO 1656, 2019) was used to convert the nitrogen content into protein, as most proteins contain 16% nitrogen. The nitrogen and protein calculations were performed according to Equations (1) and (2).

Considering $meq_{N}$ = 0.014 *g*,

- $H_{2}{\mathrm{SO}}_{4}$ = Concentration of the standardized sulfuric acid solution;
- $V_{H_{2}{\mathrm{SO}}_{4}}$ = Volume used in the titration.

The mass of nitrogen (*g*) is defined as

(1) $$N\left(g\right)=meq_{N}\times N_{H_{2}SO_{4}}\times V_{H_{2}SO_{4}}.$$

Furthermore, the protein percentage in the sample (%) is defined as

(2) Protein(%)=N(%)×FC,

where FC is the nitrogen-to-protein conversion factor (6.25).

### 2.2. Obtaining the Latex Serum

The latex serum was obtained using a procedure based on literature [19]. The latex (natural and pre-vulcanized) was diluted in distilled water at a 1:1 ratio and kept under stirring at room temperature (approximately 25 °C) for exactly 10 min using a magnetic stirrer (model C-MAG HS 7; IKA, Guangzhou, China) for homogenization.

The solution was then centrifuged in a Megafuge X3 FR centrifuge (Thermo Fisher Scientific, USA) for 1 h at 25,825× *g* and 10 °C. After this procedure, the serum fraction, as the intermediate fraction of the flask shown in Figure 1, was collected using a spatula and a syringe. The collected serum was subjected to a second centrifugation for 30 min under the same conditions. The supernatant obtained from this centrifugation was discarded.

The remaining serum was subjected to dialysis in a 25 mm × 16 mm dialysis bag with a pore size of approximately 10,000 Daltons for 72 h in a cold chamber at 8 °C, with water changes every 6 h. The pH was adjusted to 9 at the end of the procedure. Portions of serum obtained from natural latex (Batch 1) and pre-vulcanized latex (Batches 1 and 2) were stored under refrigeration (4 °C) or freezing (−20 °C) for nitrogen content determination, protein concentration, protein precipitation, and initial protein fractionation tests (only with pre-vulcanized latex serum). From Batch 3 of the pre-vulcanized latex serum, 3.0 mL aliquots of the entire obtained volume were made and immediately stored at −20 °C for later fractionation. The process yield calculation was performed using Equation (3):

(3) $$\eta =\frac{V_{ob}}{V_{in}},$$

where $V_{ob}$ is the volume of the serum obtained and $V_{in}$ is the initial volume of latex.

### 2.3. Obtaining the Protein Precipitate

The protein precipitate was obtained using a methodology based on the works of Marciano (2017) [21] and Habib and Ismail (2021) [22]. To the latex serum, a chilled solution at −20 °C was added in a 1:1 ratio, consisting of acetone, 20% (*v*/*v*) trichloroacetic acid, and 4 mmol/L dithiothreitol (DTT). This mixture was homogenized and precipitated for 12 h at −20 °C, then subjected to centrifugation at 18,000× *g* for 30 min at 4 °C.

The supernatant was discarded, and the precipitate was washed three times with a solution of acetone and DTT (10 mL of acetone at −20 °C containing 2 mmol/L of DTT). Afterward, the mixture was centrifuged again at 18,000× *g* and 4 °C for 15 min. The precipitate was then re-suspended in dimethyl sulfoxide (DMSO).

### 2.4. Protein Fractionation of the Serum

The process of protein fractionation to obtain the fraction of interest was based on the work of Morais (2017) [23] and adapted to the liquid-phase chromatography system for protein separation (ÄKTATM pure; GE Healthcare, Glattbrugg, ZH, Switzerland).

The methodology is based on the use of an anion exchange resin—namely Diethylaminoethyl Sepharose (DEAE Sepharose)—in a chromatographic column for fractionation through the interaction of the fractions with the stationary phase (resin) and the action of buffer solutions with a saline gradient. The latex serum was used to obtain the fractions, as reported in literature [23].

The HiTrap DEAE Sepharose Fast Flow 1 mL column ( Cytiva, Marlborough, MA, USA) was used for separation and equilibrated with an ammonium bicarbonate ($NH_{4}HCO_{3}$) buffer solution at 0.01 mol/L with pH adjusted to 7.8. For elution, a sodium chloride (NaCl) solution at 1.5 mol/L in $NH_{4}HCO_{3}$ 0.01 mol/L, also with pH 7.8, was employed. All solutions were filtered using a 0.22 μm filter and placed in an ultrasonic bath for 20 min to remove dissolved gas.

The selection of NaCl gradient concentrations was based on optimizing protein fractionation efficiency, ensuring high resolution and minimizing non-specific interactions. The methodology followed the principles of Morais [23], adapted to the liquid-phase chromatography system for protein separation. Specifically, a NaCl gradient ranging from 250 to 1500 mmol/L (0.25 to 1.5 mol/L) was employed to gradually elute bound proteins from the DEAE Sepharose resin. These concentrations were chosen based on the expected ionic interactions between the latex serum proteins and the anion-exchange matrix, ensuring optimal separation of the fractions of interest. The selection was further supported by previous studies and preliminary optimization trials, which demonstrated that this range allowed for effective protein recovery while preventing excessive overlap between fractions.

A volume of 3.0 mL of latex serum was added to 3.0 mL of the $NH_{4}HCO_{3}$ buffer solution. The mixture was homogenized and filtered through a 0.22 μm filter before injection into the equipment. A gradient of 16%, 33%, and 100% of the NaCl solution in $NH_{4}HCO_{3}$ was applied to separate the fractions, corresponding to concentration gradients of 0.25, 0.5, and 1.0 mol/L, respectively.

The elution was carried out at a flow rate of 1.0 mL/min with 10 column volumes (10 mL) for each elution step. The fractions were collected in Falcon^®^ tubes in 5.0 mL aliquots using an automatic collector, and the absorbance of the fractions at a wavelength of 280 nm was monitored on the equipment. Identification of the fractions was performed by comparing the chromatographic profile obtained with data from literature [23,24].

### 2.5. Characterization of the Fractions

The characterization of the fraction of interest was performed through protein concentration determination using the Bradford assay and evaluation of the electrophoretic profile on SDS-PAGE gel. The serum was subjected to the same characterization procedures as FrHB1, with the addition of total nitrogen determination using the Kjeldahl method and elemental analysis. The protein precipitate was also analyzed using the Bradford assay and the Kjeldahl method. However, several challenges were encountered during the characterization process. Protein quantification showed variations between samples, possibly due to losses during purification and fractionation steps. Additionally, during SDS-PAGE analysis, faint or undetectable bands were observed, making result interpretation difficult. These limitations may be attributed to factors such as low protein concentration, partial protein degradation, losses during dialysis and precipitation steps, and limitations in gel staining sensitivity. To mitigate these challenges, additional strategies were implemented, including pre-concentration of samples using SpeedVac, optimization of sample loading in the gel, and testing alternative staining methods to improve protein detection.

#### 2.5.1. Bradford Assay

In this assay, 20 μL of the samples were mixed with 180 μL of Coomassie Blue Protein Assay dye (Thermo Fisher Scientific, USA). An analytical curve with 7 points (0.01 to 0.2 mg/mL) was constructed using the BSA (Pierce™ Bovine Serum Albumin) standard (Thermo Fisher Scientific, USA). The absorbance of the samples and the analytical curve were measured at a wavelength of 595 nm at 25 °C using the Varioskan Lux multimode microplate reader (Thermo Fisher Scientific, USA). The protein concentration in the samples was calculated using the equation of the line obtained from the analytical curve.

#### 2.5.2. Electrophoresis

Characterization via electrophoresis was carried out using BoltTM 4–12% Bis-Tris-Plus gel (Invitrogen, Waltham, MA, USA, Thermo Fisher Scientific Inc., Waltham, MA, USA). Laemmli buffer was prepared from 10 mL of 1.0 M Tris (pH 6.8), 4.0 g of SDS, 20 mL of Glycerol, 10 mL of β-Mercaptoethanol, 0.1 g of Bromophenol Blue, and ultrapure water up to 50 mL (COLD SPRING HARBOR PROTOCOLS, 2015). To 15 μL of the samples, 3.75 μL of the Laemmli buffer (heated to 96 °C for 10 min) was added. The run was performed with 20X Bolt MES SDS Running Buffer (Thermo Fisher Scientific, USA) at 200 V for 25 min using the Mini gel tan (Invitrogen, Thermo Fisher Scientific, USA) and EnduroTM power supplies 250 V (Labnet, Edison, NJ, USA). The reference used was the SeeBlue Plus 2 PreStained Standard (Invitrogen, Thermo Fisher Scientific, USA), and the gel was stained with Fast Blue Stain Reagent (Scienco, São Paulo, SP, Brazil) with the addition of 0.125 g of potentiator (Scienco, Brazil) to speed up the staining process. Gel visualization was performed using the ChemiDocTM MP Imaging system (Bio-Rad, Hercules, CA, USA).

## 3. Results

### 3.1. Characterization of Latex

The two types of latex (natural and pre-vulcanized) were evaluated for their characteristics. The natural latex had a more whitish appearance, a creamier texture, and a strong ammonia odor. On the other hand, the pre-vulcanized latex had a more yellowish tone, a more liquid texture, and a lighter odor. The protein content was calculated for both types of latex through elemental analysis and the Kjeldahl method. Table 1 presents the obtained results. To calculate the protein content from the nitrogen content, a conversion factor of 6.25 was used, which is commonly applied to plant and food samples. One of the reasons for overestimation of the protein content observed for pre-vulcanized latex using the Kjeldahl method is the presence of nitrogen in non-protein compounds, such as urea, ammonia, or nucleic acids. These compounds are hydrolyzed and converted into ammonia during digestion, and are then included in the total nitrogen measurement, leading to the overestimated protein content.

### 3.2. Serum

**Figure 4:**
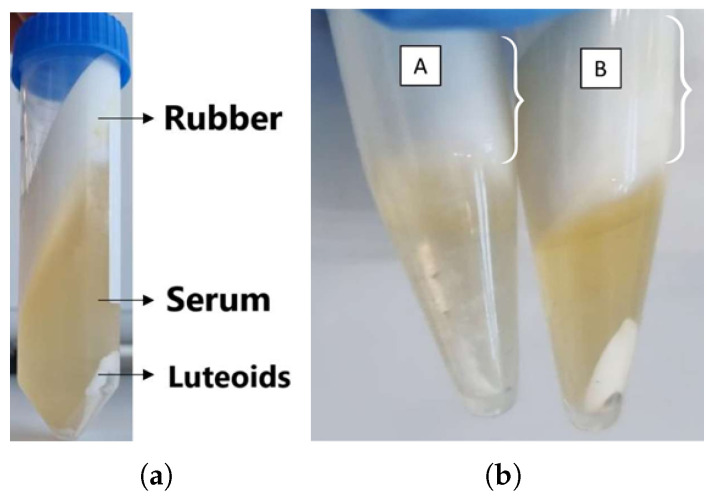
(**a**) Distinct fractions of raw latex after centrifugation, showing the separation into three layers: rubber (upper), serum (intermediate), and lutoids (lower); (**b**) visual appearance of the phases of natural latex (A) and pre-vulcanized latex (B) after centrifugation. Source: the authors, 2024.

The initial serum extraction tests were based on a methodology using acetic acid, which was not efficient in obtaining the serum. The process took 3 days, with the sample exposed to room temperature, and the resulting serum had a high concentration of acetic acid. Therefore, the alternative methodology described in Section 2.2 was implemented. This procedure allowed for separation of the three characteristic phases reported in the literature, with the serum being the intermediate fraction (Figure 4a).

After centrifugation, a qualitative comparison of the visual appearance of each latex was made. The serum from natural latex was clear (Figure 4b, A), with a denser and more solid rubber and a smaller portion of lutoids. The serum from pre-vulcanized latex (Figure 4b, B) was more yellowish, the rubber was pasty and difficult to separate, and it contained a larger portion of lutoids compared to natural latex.

The serum was dialyzed for further purification of the sample. The nitrogen content was calculated for the serum obtained from natural latex and pre-vulcanized latex (Table 2). The protein content was not determined by nitrogen quantification methods due to uncertainty regarding the conversion factor for proteins, which could result in either overestimated or underestimated value of the actual protein concentration in the samples. Only the serum obtained from pre-vulcanized latex was used for the subsequent procedures of protein precipitate acquisition and protein fractionation.

**Figure 5:**
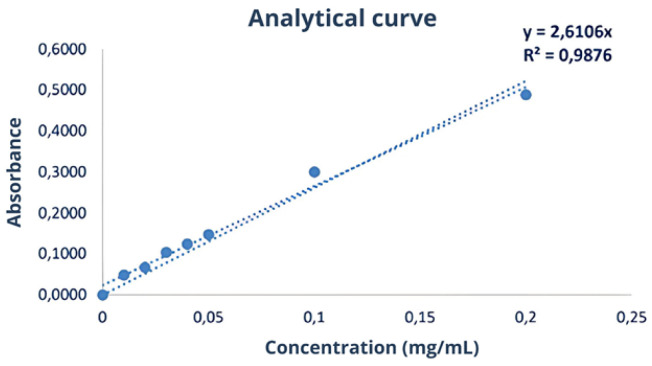
Analytical curve of the first Bradford assay used to calculate protein concentrations in serum, illustrating the relationship between absorbance and protein concentration. This curve was constructed using standard solutions of Bovine Serum Albumin (BSA) at concentrations ranging from 0.01 to 0.2 mg/mL. Each standard solution was mixed with the Bradford reagent, and absorbance was measured at 595 nm. Absorbance values were plotted against known concentrations to generate the standard curve. A linear regression analysis was performed to derive the equation of the line, which allows the determination of protein concentrations in unknown samples by interpolating their absorbance values. Source: the authors, 2024.

The yield of the first batch of this serum was 40.62%. The protein concentration for the first serum batch was determined using the Bradford assay and calculated as 1.52 ± 0.06 mg/mL. However, statistical significance measures, such as *p*-values, were not provided in this context. The study did not conduct statistical comparisons between different batches or experimental conditions at this stage, as the focus was on determining protein concentration rather than hypothesis testing. Additionally, the protein content was not determined using nitrogen quantification methods due to uncertainties in conversion factors. A 10-fold dilution was applied, and the concentration calculated for the serum using the equation is derived from Figure 5.

**Figure 6:**
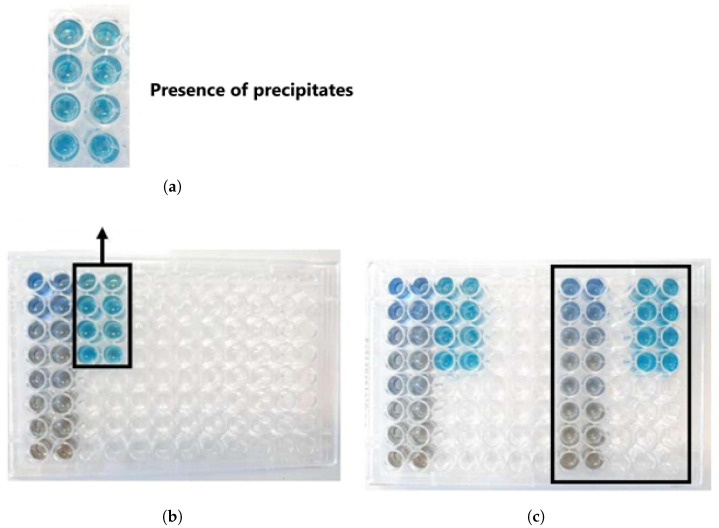
Bradford assay for determining protein concentrations in Batches 1 and 2 of latex serum. (**a**) First assay; (**b**) Indication of precipitate formation; and (**c**) Second assay, in which the reduction of incubation time eliminated the precipitate formation observed in the previous step (b), resulting in a clearer solution. The x-axis is adjusted to reflect the BSA concentration units in the analytical standard (0.01 to 0.2 mg/mL), as described in the Bradford assay methodology. described in the Bradford assay methodology. The first peak in the chromatogram corresponds to the first eluted substance, which is a protein of interest in the chromatography process. This peak is identified and now properly described in the figure caption. Source: the authors, 2024.

A second batch of serum was prepared, with an approximate yield of 49.74%. A new Bradford assay was performed to determine the concentrations in the serums (Batches 1 and 2; Figure 6b). After resting with the reagent for more than 5 min, precipitates were observed (Figure 6a). The experiment was repeated by reducing the incubation time, which eliminated the occurrence of precipitation (Figure 6c). In this assay, no dilution factor was applied to the serum samples, resulting in absorbances higher than the upper limit of the analytical curve. Therefore, the protein concentration was not calculated to avoid errors in estimation due to extrapolation of the curve. Thus, Table 3 presents only the absorbance results found through this assay.

**Figure 7:**
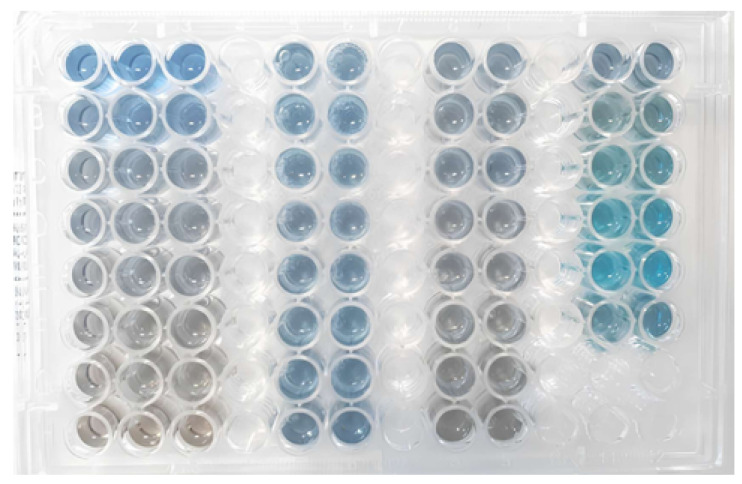
Bradford assay for determining protein concentration in the third serum batch and fractions obtained from Fractionations 5 to 9. Source: Authors, 2024.

**Figure 8:**
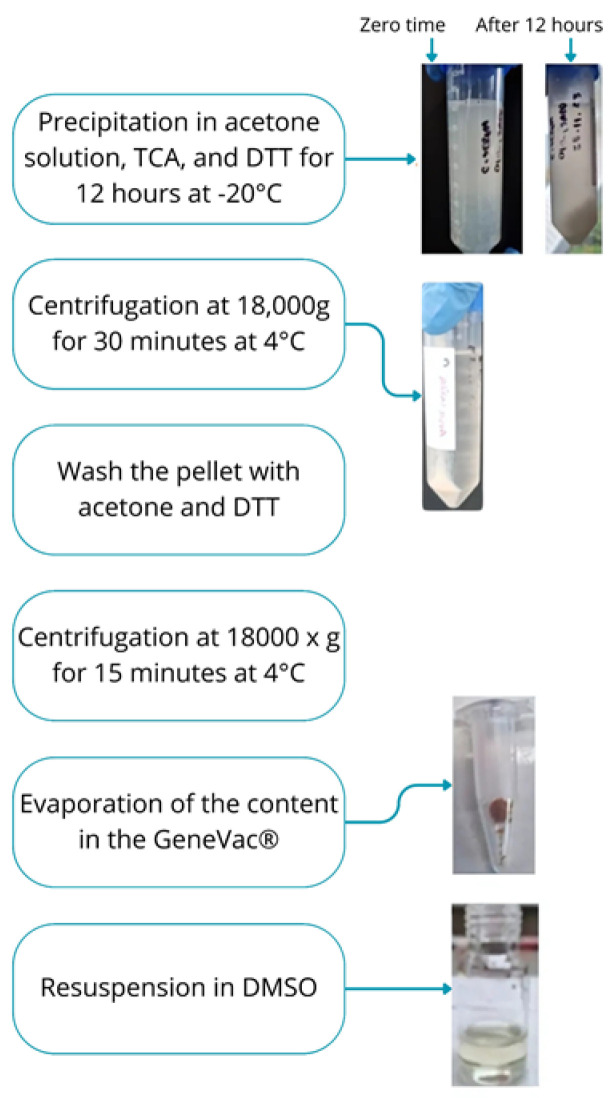
Flowchart of the precipitation process. Reagents: TCA = trichloroacetic acid, DTT = dithiothreitol, and DMSO = dimethyl sulfoxide. Source: the authors, 2025.

**Figure 9:**
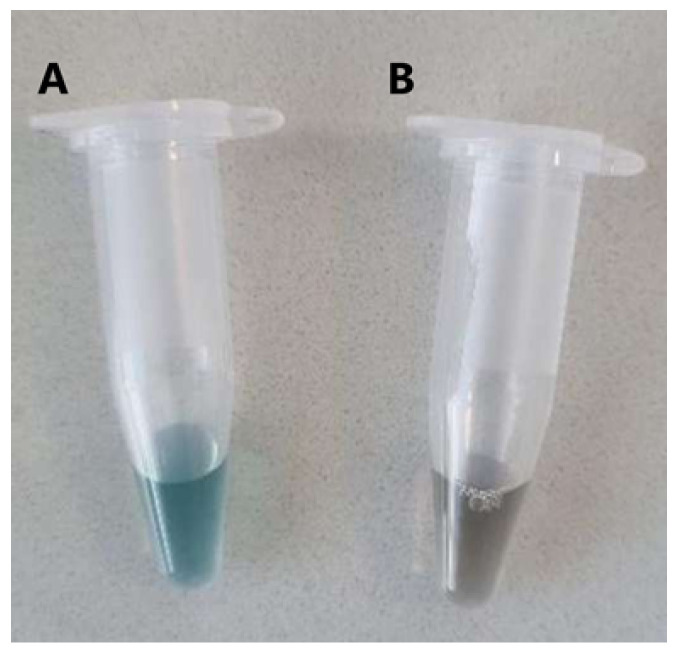
Test to observe the tolerance of the colorimetric reagent with the DMSO dilution: (**A**) diluted and (**B**) pure. Source: the authors, 2024.

**Figure 10:**
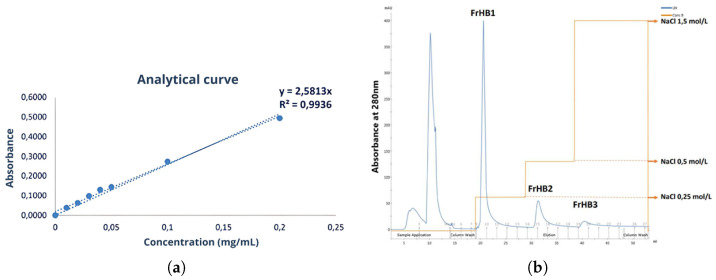
(**a**) Analytical curve of the Bradford assay used to calculate protein concentrations in the third serum batch and the fractions obtained from Fractionations 5 to 9, showing the relationship between absorbance and protein concentration; (**b**) chromatographic profile of the latex serum fractionation obtained with the adaptation of the methodology to the ÄKTA system. The main fractions—FrHB1, FrHB2, and FrHB3—were obtained with NaCl gradients at concentrations of 0.25 mol/L, 0.5 mol/L, and 1.5 mol/L, respectively. Source: the authors, 2024.

A third batch of serum was obtained with a yield of 57.19%. The protein concentration was determined using the Bradford assay (Figure 7). Figure 8 presents the flowchart of the precipitation process, while Figure 9 shows the test to assess the tolerance of the colorimetric reagent to the DMSO dilution. The protein concentration was calculated by applying the equation derived from the analytical curve shown in Figure 10a. Using a 10-fold dilution for the assay, the protein concentration in the serum was found to be 1.8477 ± 0.0336 mg/mL.

### 3.3. Protein Precipitate

The protein precipitation procedure (Figure 8) was performed with the aim of confirming the presence of proteins in the serum and evaluating the possibility of obtaining the FrHB1 fraction from the extract, as the precipitate has a higher concentration of proteins and fewer interfering components for separation.

The choice of the protein precipitation method using trichloroacetic acid and acetone was based on references reporting the best yield of this method compared to others as well as better visualization in two-dimensional electrophoresis gels, facilitating proteomic studies of latex serum [21,25].

The method required adaptations due to some specific characteristics of the sample. One of the main challenges occurred during the second centrifugation step. In the samples in question, a sufficiently structured pellet was not formed for the supernatant to be discarded. Therefore, to avoid protein loss, a solvent removal process was carried out using a GenVac^®^ device that controls rotation, temperature, and pressure. This resulted in a brown-colored precipitate, as shown in Figure 9. The reference method involved re-suspending the precipitate in water; however, the precipitate from this type of latex was not soluble in water. An alternative for re-suspension in water is solubilization in an alkaline solution; however, the sample still remained insoluble. Finally, solubilization in DMSO PA was tested, in which the precipitate became soluble.

In this precipitation phase, the necessary adjustments to the method made the subsequent steps more difficult. The quantification in the Bradford assay also required adaptation, as the method only tolerates up to 10% DMSO. Using the dilutions for protein quantification, a concentration of 2.357 ± 0.231 mg/ml was obtained by applying the equation derived from Figure 10a. Therefore, to facilitate the separation of the fraction, the method was developed directly from the latex serum, as precipitation involves steps that subject proteins to stress, potentially altering their structure. Moreover, methodological references for fractionation use latex serum as the starting material.

### 3.4. Protein Fractions and Characterization

The fractionation methodology was adapted to be carried out on automated equipment, requiring development and optimization steps. It was agreed with the client, in February of 2024, that the volume of the fraction to be delivered would be 70 mL, and this became the target to be achieved.

#### 3.4.1. Fractionation 1

**Figure 11:**
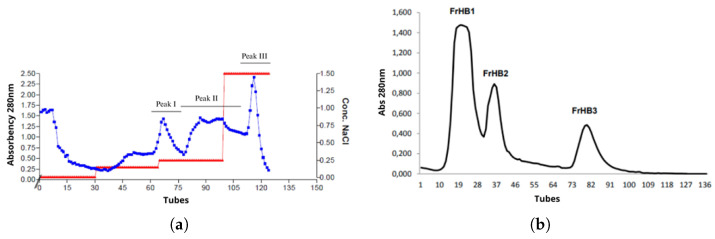
Reference for the chromatographic profile of the latex serum fractionation. Source: (**a**) Profile obtained by Souza (2007), showing three main peaks (Peak I, Peak II, and Peak III) with corresponding NaCl concentrations; (**b**) fractionation profile identified by Morais (2017), with three main fractions: FrHB1, FrHB2, and FrHB3.

Due to limitations of the column used regarding the amount of protein that could be applied, the initial tests adapting the reference methodology to the ÄKTA system were conducted by introducing 1.0 mL of latex-extracted serum. Each elution step consisted of 10 column volumes (10 mL) with the corresponding saline gradient solution mixture. Collections were made in Falcon tubes in 2.0 mL increments. The chromatographic profile obtained (Figure 10b through UV absorbance monitoring) was consistent with that observed in the literature (Figure 11a,b). The FrHB1 fraction—the target of this study—was observed to elute in the first stage of separation with a NaCl concentration of 0.25 mol/L.

**Figure 12:**
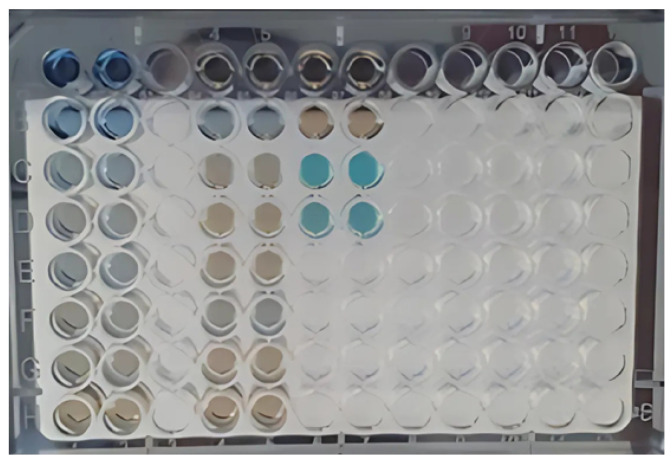
Bradford assay for determining protein concentrations in the obtained fractions. Source: the authors, 2024.

**Figure 13:**
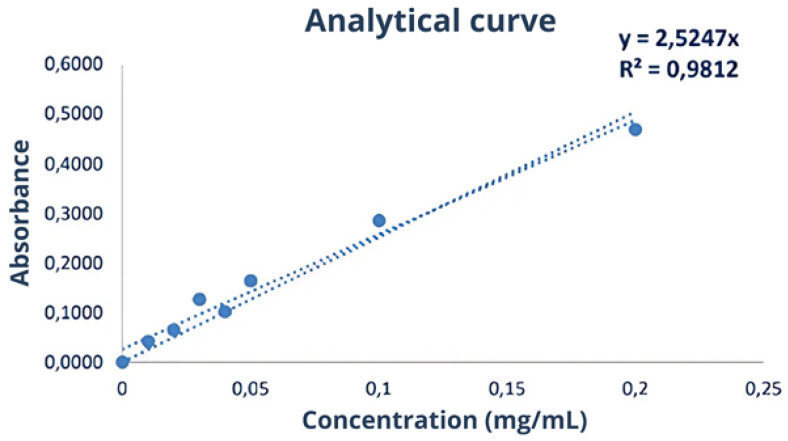
Analytical curve of the Bradford assay used to calculate protein concentrations, showing the relationship between absorbance and protein concentration. Source: the authors, 2024.

The Bradford assay was used to determine the protein concentration in each of the collected fractions. In Figure 12, the plate containing the standards of the analytical curve, fractions collected in the first fractionation assay, and raw serum (all stained with Coomassie blue dye) are presented. The absorbance corresponding to each well was measured, and the concentration was calculated using the equation of the line obtained from the analytical curve (Figure 13).

The protein concentrations in each collected vial are presented in Table 4. The FrHB1 fraction showed the highest average concentration, yielding approximately 0.876 mg of the fraction from 1.0 mL of serum (Table 4).

**Figure 14:**
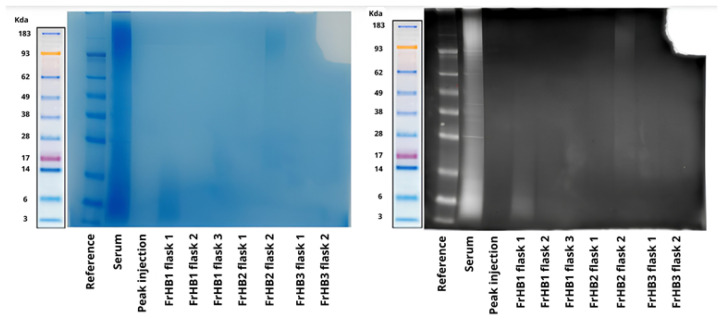
Electrophoretic profile on Bolt gel after the first fractionation in the ÄKTA system with samples from the fractions collected from FrHB1 and FrHB2. The protein distribution is observed according to molecular weight in kDa. Source: the authors, 2024.

**Figure 15:**
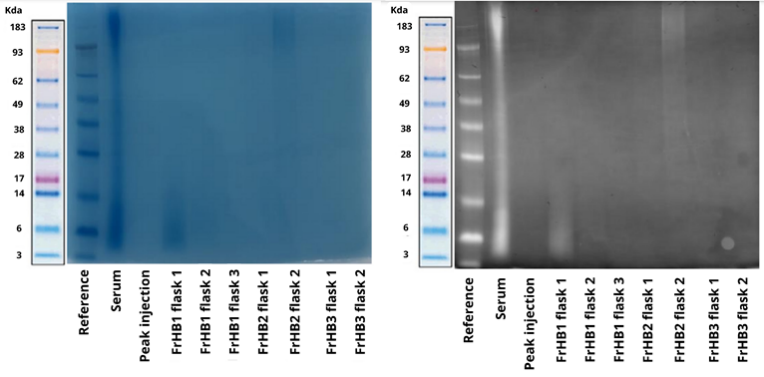
Electrophoretic profile on Bolt gel after the first fractionation in the AKTA system with samples pre-concentrated using SpeedVac before gel application. The molecular weight reference (kDa) indicates protein sizes for comparison. Source: the authors, 2024.

To confirm the size of the proteins present, SDS-PAGE was performed. Figure 14 shows the result of the electrophoresis. Smooth bands were observed for the latex serum; however, no bands were visible in the obtained fractions. Suspecting that the protein concentration was below the sensitivity of the gel, a pre-concentration of the fractions was performed using a Savant DNA 120 SpeedVac concentrator (Thermo Fisher Scientific, USA) followed by a repeat experiment, which yielded the same result (Figure 15).

#### 3.4.2. Fractionation 2

**Figure 16:**
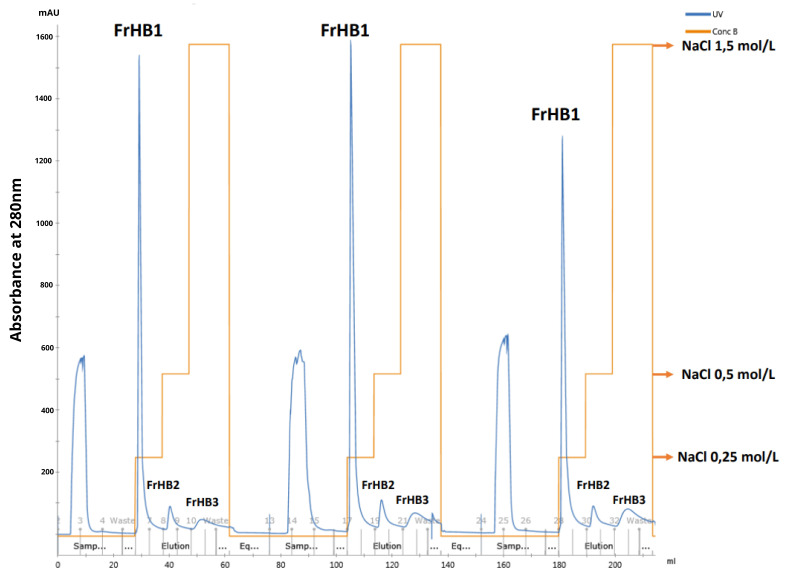
Chromatographic profile obtained in the second latex serum fractionation test. The graph shows absorbance at 280 nm (blue line) as a function of volume (ml), with three elution stages performed at different NaCl concentrations (0.25 mol/L, 0.5 mol/L, and 1.5 mol/L). Source: the authors, 2024.

In the subsequent fractionations, the second batch of dialyzed serum (stored under freezing conditions) was used, and the injected volume into the column was increased to 2.0 mL while maintaining the elution conditions and increasing the collection volume to 5.0 mL in order to obtain the entire peak of interest in the same tube. The purpose of increasing this volume was to enhance the protein concentration in the collected fraction. In this test, two consecutive runs were performed, and the same chromatographic profile was observed in both, confirming the robustness of the method employed (Figure 16).

**Figure 17:**
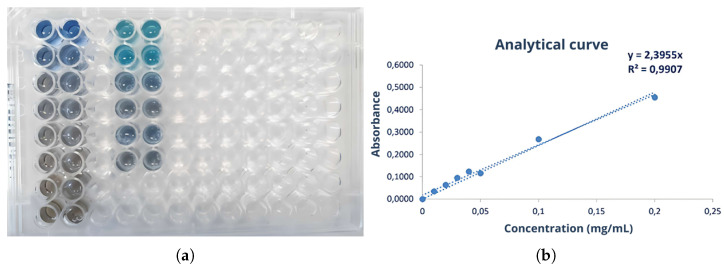
(**a**) Bradford assay for determination of protein concentration in the fractions obtained from the second fractionation; (**b**) analytical curve of the Bradford assay used to calculate protein concentrations in the second fractionation, showing the relationship between absorbance and protein concentration. Source: the authors, 2024.

The Bradford assay was performed to determine the protein concentration in the fractions (Figure 17a,b, and the obtained concentrations are presented in Table 5). FrHB3 was excluded from the quantification due to its low peak intensity.

**Figure 18:**
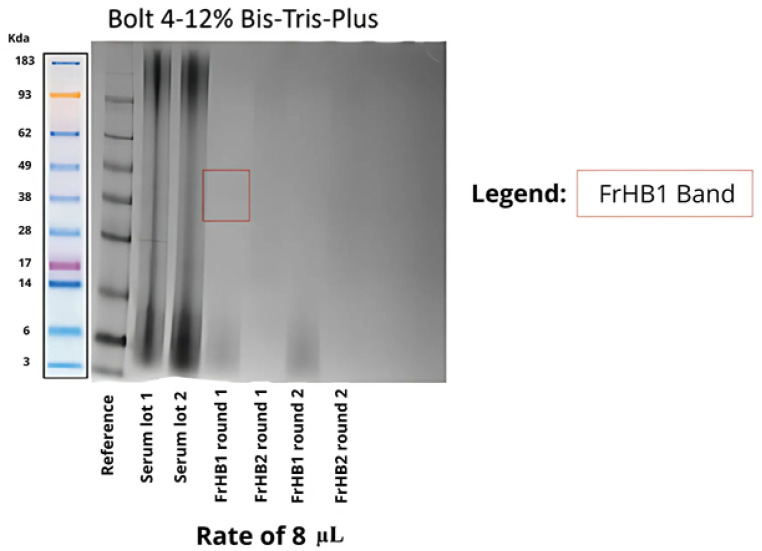
Electrophoretic profile of fractions obtained from the second latex serum fractionation (Batch 2) using Bolt 4–12% Bis-Tris-Plus gel and applying 8 μL of each sample. The distribution of proteins is shown according to molecular weight, with emphasis on the expected band for the FrHB1 protein. Source: the authors, 2024.

In the analysis of the electrophoretic profile, a faint band was observed for the FrHB1 fraction obtained from the second batch of serum fractionation; meanwhile, for the second run, no band was visualized for FrHB1 (Figure 18). A scheme of different concentrations was employed for further evaluation of the electrophoretic profile on a gel of different composition (NuPAGETM 4–12% Bis-Tris). The serum and the obtained fractions were pre-concentrated in SpeedVac and subjected to dilutions.

**Figure 19:**
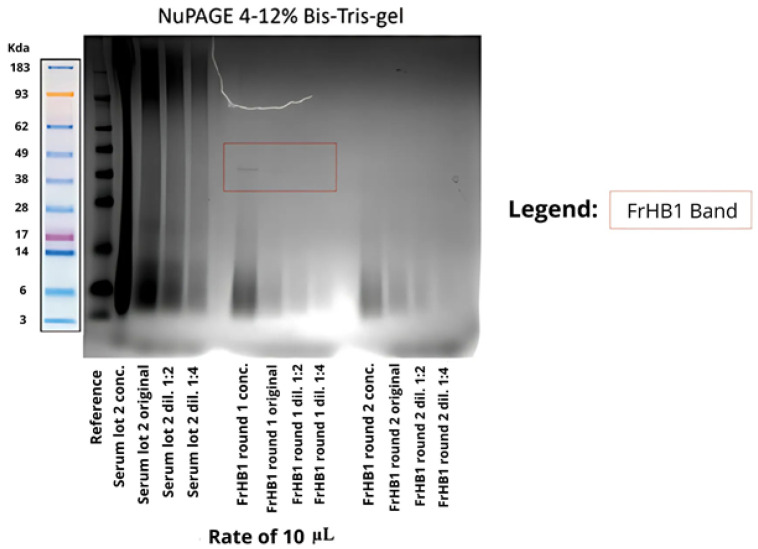
Electrophoretic profile of the fractions obtained after the second serum fractionation in NuPAGE 4–12% Bis-Tris gel, showing the protein distribution according to molecular weight. The samples were applied at 10 μL each, and the dilutions indicate different concentrations and dilutions of the analyzed fractions. The marked band represents the expected location of the FrHB1 protein. Source: the authors, 2024.

The concentrations in each analyzed sample are described in Table 6. Figure 19 shows the profile obtained from the new gel, whose run lasted 35 min.

It is possible to observe the intensification of the band seen in FrHB1 obtained in the first round of fractionation with to the bestin sample concentration applied to the gel. The electrophoretic profile of the serum is diffuse, and visualization is reduced in the most concentrated sample for which a pronounced trailing effect can be observed.

#### 3.4.3. Fractionation 3

**Figure 20:**
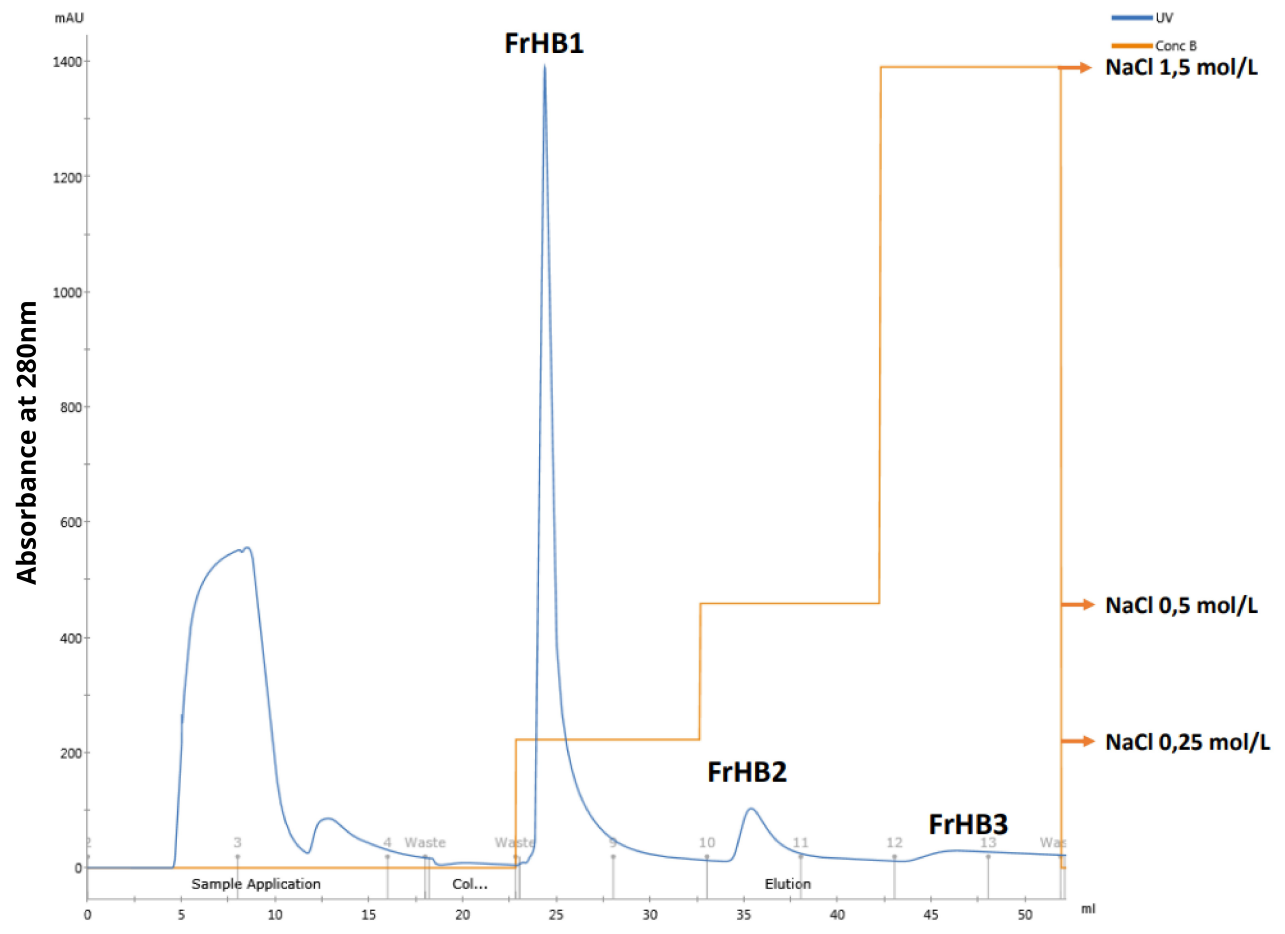
Chromatographic profile obtained in the third latex serum fractionation test with the addition of PMSF. The graph shows absorbance at 280 nm (blue line) as a function of volume (mL), with three elution steps performed with different NaCl concentrations (0.25 mol/L, 0.5 mol/L, and 1.5 mol/L). Source: the authors, 2024.

**Figure 21:**
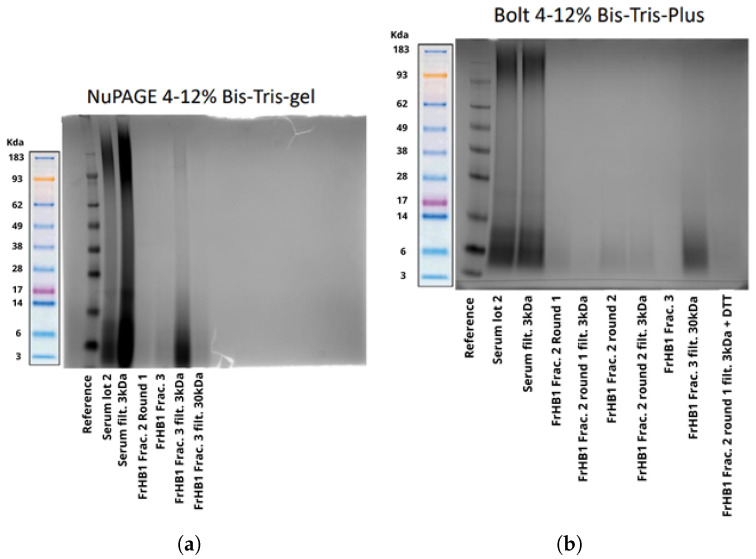
Electrophoretic profile of the fractions obtained in the second and third ÄKTA fractionation experiments, with filtration in Amicon and the addition of DTT. The images show the use of two types of gels in the electrophoresis experiment: NuPAGE 4–12% Bis-Tris gel (**a**) and Bolt 4–12% Bis-Tris Plus gel (**b**). The bands represent the fractions and conditions tested, with their respective molecular weights indicated in kDa. Source: the authors, 2024.

In the next experiment, a protease inhibitor was added to the buffers and serum before fractionation in order to assess whether the stability of the proteins in the fraction of interest would be increased. Upon addition, a precipitate formed in the buffers and serum mixed with the buffer. The solutions and sample were filtered before being introduced into the ÄKTA system to avoid clogging. In this experiment, 3.0 mL of serum from Batch 2 was used, and the column was kept refrigerated. The chromatogram presented the same profile as the previous ones (Figure 20), with the fractionation not being influenced by precipitation. Electrophoresis experiments were performed both with NuPAGE gel (Figure 21a) and Bolt gel (Figure 21b). Filtration of the fractions in Amicon^®^ 3 kDa and 30 kDa was tested to pre-concentrate the samples, and Dithiothreitol (DTT) was applied to stabilize the fraction and allow for better visualization of the bands. The FrHB1 fractions obtained in the previous test (Rounds 1 and 2) and in the current test were evaluated; however, no bands were observed in the fractions.

#### 3.4.4. Fractionation 4

**Figure 22:**
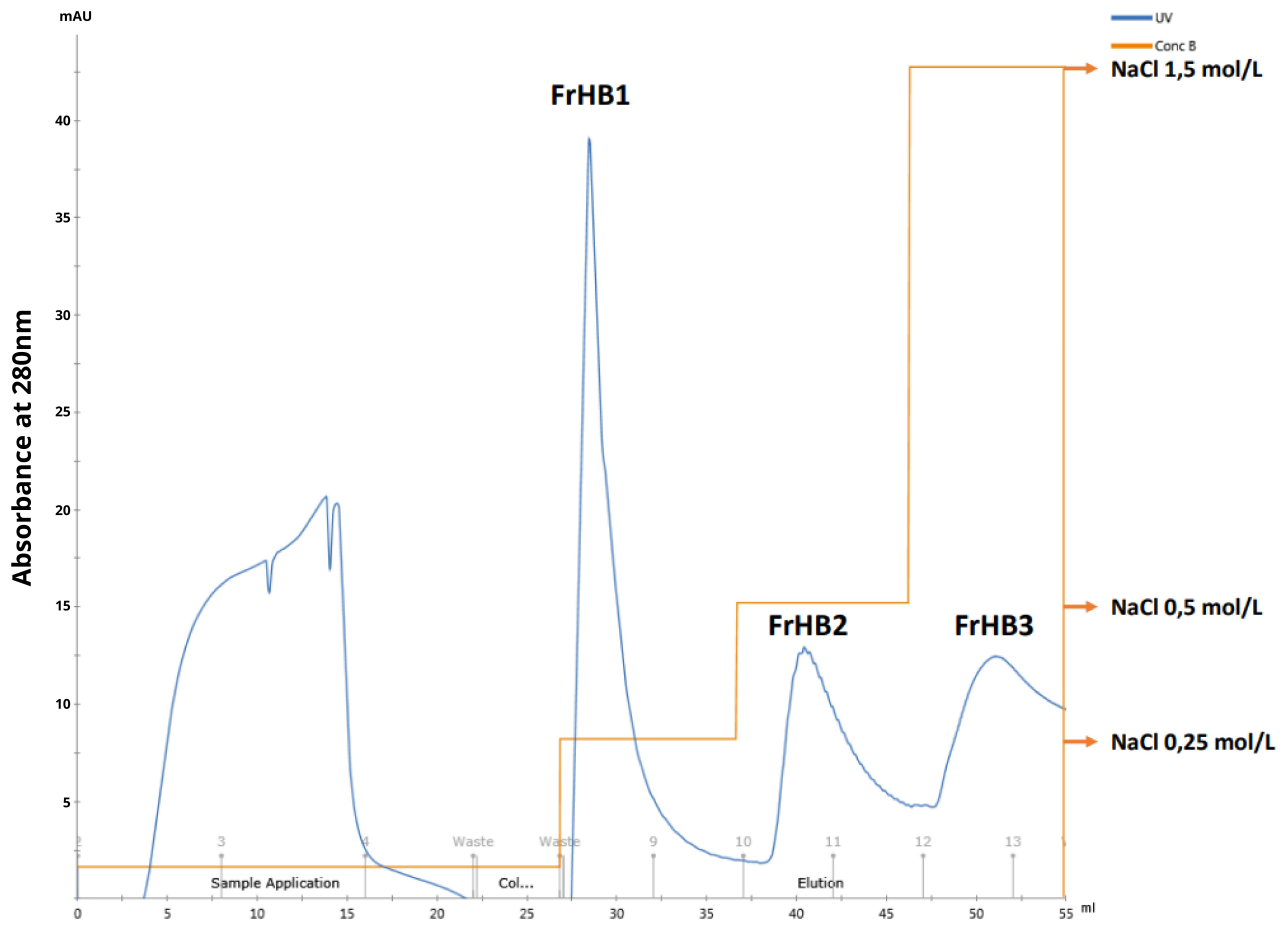
Chromatographic profile of the fourth latex serum fractionation test, after re-injection of the sample. The graph shows absorbance at 280 nm (blue line) as a function of volume (mL), with three elution steps carried out at different NaCl concentrations (0.25 mol/L, 0.5 mol/L, and 1.5 mol/L). Source: the authors, 2024.

Another fractionation procedure was carried out using Batch 2 of latex serum. No protease inhibitor was used, and the solutions were kept in an ice bath throughout the experiment. Again, 3.0 mL of Batch 2 serum was used in the experiment. Due to a failure during the sample injection step, the expected profile was not observed in the chromatogram. The sample was then re-injected, producing the expected profile for the chromatogram (Figure 22). Electrophoresis was performed on Bolt gel with an increase from 15 μL to 60 μL in the sample volume loaded into the wells. No bands were observed in the experiment.

#### 3.4.5. Fractionations 5 to 9

To avoid the influence of denaturation, a third batch of serum and new buffer solutions were prepared for the following experiments. The solutions were stored at 4 °C under refrigeration. The serum was separated into 3.0 mL aliquots immediately after production and kept frozen at −20 °C until fractionation. The serum was thawed under refrigeration (4 °C) to minimize the impact of temperature changes. The solutions were maintained in an ice bath to ensure low temperatures throughout the sample path. The collected fractions had 200 μL aliquots, immediately separated for characterization experiments, and both the aliquots and original fractions were stored at −20 °C.

**Figure 23:**
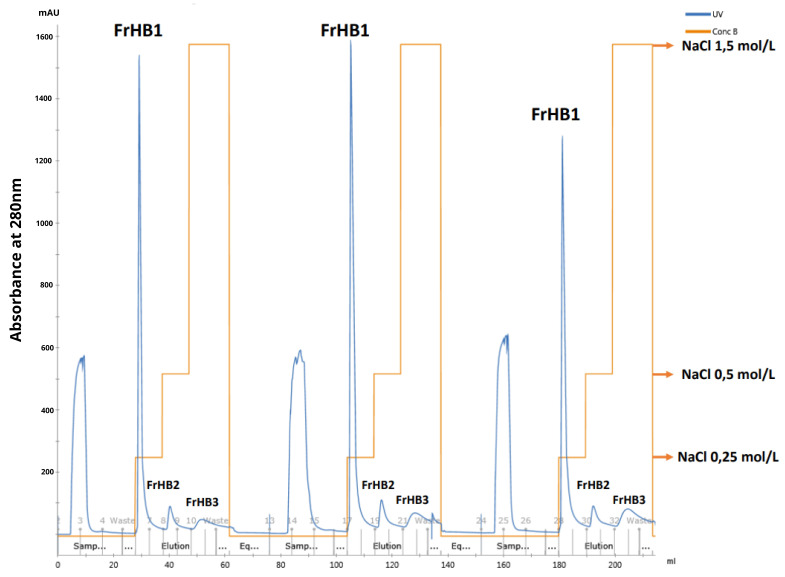
Chromatogram of the fifth fractionation of latex serum for obtaining the fractions of interest. The graph shows absorbance at 280 nm (blue line) as a function of volume (mL), with three elution steps at different NaCl concentrations (0.25 mol/L, 0.5 mol/L, and 1.5 mol/L). Source: the authors, 2024.

In each experiment, three or five fractionation runs were performed to produce the fraction of interest. The chromatographic profile remained consistent, as shown in Figure 23. A decrease in the intensity of the FrHB1 peak was observed in the third run, suggesting possible column saturation. The low capacity of the column (1 mL) made it challenging to obtain the desired fraction through consecutive serum fractionation. The need to use this column arose from a delay by the supplier in shipping the purchased material for preparation of a larger-capacity column, which hindered the initial plan. Column cleaning runs were necessary between fractionation steps to maintain, as much as possible, the separation capacity.

A total of 17 fractionation rounds were carried out with the third batch of serum under the conditions described in this section, resulting in a total of 70 mL of the fraction of interest. To determine the protein concentration in the fractions, a final Bradford assay was performed (Figure 7). The analytical curve (Figure 10a) and the calculated concentrations (Table 7) are presented below.

The decrease in concentration observed in the final rounds was consistent with the saturation of the chromatographic column due to the frequency of use and the high protein load and other compounds introduced into the column. This saturation, however, did not hinder the fulfillment of the agreement regarding the required fraction amount and allowed for maintenance of the expected average concentration.

## 4. Discussion

The historical context of the use of latex and studies into its potential biomedical properties spans back to the middle of the late 14th century, when Christopher Columbus brought it to Europe from America. Then, in 1839, Charles Goodyear developed the vulcanization technique, which enabled exorbitant growth in the automobile industry until it became the world’s largest exporter of natural rubber in 1890. Due to its great genetic variability and the production of different clones, the productivity of rubber plants is widespread. Seed smuggling in 1877 to countries such as Malaysia, England, and Ceylon led to the end of the Brazilian monopoly. At present, 90% of the global production of rubber takes place in Asian countries [26].

Against this backdrop of the evolution and application of latex, the results obtained regarding the characterization of natural and pre-vulcanized latex revealed significant differences in physical and chemical properties between these two types of latex. Natural latex showed a protein content of 3.49 g/100 g via elemental analysis and 3.05 g/100 g when using the Kjeldahl method, while pre-vulcanized latex showed 3.37 g/100 g via elemental analysis and 3.95 g/100 g via the Kjeldahl method (Table 1). These data indicate that the protein content is pre-vulcanized latex may be overestimated, possibly due to the presence of non-protein nitrogen compounds (e.g., urea and ammonia) which are released during digestion, thus affecting the accuracy of Kjeldahl measurements.

Several fractionations were carried out in order to study the protein fractions from natural and pre-vulcanized latex for various reasons, all aimed at understanding and applying the biochemical and functional properties of these proteins. Fractionation helps to reduce the presence of unwanted contaminants, such as lipids or carbohydrates, which can interfere with biological tests or the characterization of proteins. Through purifying the proteins, it can be ensured that the data obtained are more accurate and reliable. In addition, purer fractions generally have greater biological activity, allowing for a better assessment of their properties and potential for applications in the biomedical field. In this study, four fractionations were necessary to obtain a total of 70 mL of the protein fraction.

The methodology for obtaining the latex serum proved to be an initial challenge, as the use of acetic acid resulted in a low-quality serum. The alternative centrifugation approach enabled the effective separation of the latex fractions, with the serum from natural latex having a clear appearance and that from pre-vulcanized latex having a yellowish hue and a greater amount of luteoids (Figure 4a,b). The yield of the first batch of serum was 40.62%, and the protein concentration was measured at 1.52 ± 0.06 mg/mL using the Bradford assay. A second batch of serum, with a yield of 49.74%, showed absorbance values that indicated the need to optimize the method, as the concentrations could not be determined due to results lying outside the analytical curve.

The faint intensity of the band observed in the FrHbI fractions can be attributed to several factors, including the low protein concentration in the analyzed samples, which may compromise proper visualization in the SDS-PAGE gel. Additionally, the protein may undergo partial degradation during the extraction steps, further reducing the amount of intact protein available for analysis. The nature of FrHbI itself, being a plant protein, may also present challenges, such as reduced stability after extraction and structural characteristics that hinder its detection in the gel. Another factor contributing to the reduced band intensity could be limitations in staining methods or the sensitivity of the gel used, which may not be sufficient to detect proteins at very low concentrations [13,23,24].

The third batch of whey had a yield of 57.19% and a protein concentration of 1.8477 ± 0.0336 mg/mL, which suggests an improvement in the whey extraction and purification process. The protein precipitation with trichloroacetic acid and acetone was effective, resulting in a brown precipitate that, after solubilization in DMSO, had a protein concentration of 2.357 ± 0.231 mg/mL. This variation in protein concentration can be attributed to factors such as the efficiency of solubilization and the initial protein concentration in the serum. However, it is important to note that an overestimation of the initial protein content could influence the interpretation of the solubilization efficiency and the precipitation process, potentially leading to overestimated protein concentrations in the final sample. This, in turn, could affect the assessment of the extraction and purification process efficiency, as well as the reproducibility of the results across different batches.

Protein fractionation was carried out using methods adapted for the ÄKTA automated equipment, where the FrHB1 fraction was eluted in the first separation stage with 0.25 mol/L NaCl (Figure 23). The FrHB1 fraction showed the highest average concentration among the fractions obtained, with 0.180 ± 0.002 mg/mL in vial 1 and 0.135 ± 0.003 mg/mL in Vial 2 (Table 4). These data indicate the significant presence of proteins of interest in this fraction, which may be exploited for biotechnological applications.

Characterization via electrophoresis revealed significant challenges, especially with regard to the Bolt gel experiment for the fractions obtained in Fractionations 5 to 9, which resulted in no visible bands. This lack of bands can be interpreted in light of the information available in the literature, which indicates that the predominant bands in natural crude latex are of low molecular weight, with maximum intensities observed at 14 and 24 kDa [27]. The lack of visibility of these bands may be related to the nature of the proteins present in these fractions, which may be below the limit of detection of the method used or undergo changes that make it difficult to separate them effectively.

Particularly with regard to the FrHB1 fraction, there is a lack of information in the literature about its electrophoretic profile. This reinforces the complexity of samples of plant origin, which can be influenced by a variety of factors, including the genetic variability of plants, growing conditions, and extraction methods. These variables can significantly impact the composition and structure of the proteins present which, in turn, can interfere with the separation profile observed during electrophoresis.

In addition, defining more effective separation procedures would require in-depth NaCl of the proteins present in the fraction, which requires more sophisticated analytical techniques. Therefore, although electrophoresis did not provide the expected results, it is important to note that this is not the only method for confirming the presence of proteins in the fraction. The presence of proteins was corroborated by the chromatographic profile, in agreement with the existing literature, and by determining the protein concentration using the Bradford assay.

### Extraction of FrHb1 from Natural Latex

The findings on *Hevea brasiliensis* latex are in line with previous clinical studies that have indicated acceleration of the healing process when using latex biomembranes. This suggests a common underlying mechanism that can be exploited in healing therapies. The development of latex-based products, potentially enriched with the angiogenic components identified, could represent a new strategy for treating chronic wounds and other conditions requiring tissue repair.

Efforts to purify and characterize the active components of *Hevea brasiliensis* latex are fundamental to understanding its role in wound healing. Treating the serum extracted from the latex with denaturing methods, such as boiling and proteolysis, revealed the nature of its protein content, suggesting that the proteins present are responsible for increasing vascular permeability. Tests carried out on chorioallantoic membranes (CAVMs), conducted by Mendonça (2004) [13] in chicken embryos, showed that the serum stimulates angiogenesis, which is a positive indication for tissue regeneration. The initial purification of serum from RRhim 600 and GT-1 clones using a DEAE-cellulose column resulted in three fractions, with Peak I being the most effective in promoting vascular permeability and the induction of angiogenesis, as well as demonstrating, in histological studies, an increase in the thickness of the MCAs, which suggests a greater deposition of extracellular matrix. Although the second stage of purification on a CM-cellulose column did not allow us to identify which fraction was most active, the evidence points to the beneficial effects of the protein on tissue repair [13].

The difficulty in obtaining an electrophoretic profile of the FrHbI fraction can be attributed to the low concentration of the protein and interactions with the medium, which affect its mobility [28]. The low intensity of the bands in SDS-PAGE suggests limitations in detection, possibly due to the charge, size or hydration of the surface [28]. Alternative methods, such as mass spectrometry, can provide a more detailed analysis.

## 5. Conclusions

The results of this study demonstrate that *Hevea brasiliensis* latex has bioactive components with significant potential for biomedical applications, especially in wound healing. The characterization of the protein fractions, carried out using purification techniques and biological activity analyses, revealed that the proteins present in natural and pre-vulcanized latex can stimulate angiogenesis and increase vascular permeability—essential characteristics for tissue regeneration. However, the purification and characterization process faced challenges, such as overestimation of the protein content in pre-vulcanized latex and difficulty in visualizing electrophoretic bands. These limitations highlight the complexity of samples of plant origin, which can be influenced by several variables such as genetic variability and extraction methods. Therefore, a deeper understanding of the biochemical properties of latex and the optimization of separation methods is crucial.

Based on the findings, several future applications can be explored, especially regarding the use of the most bioactive fraction of latex for the development of innovative wound-healing therapies. Bioactive components identified in latex—such as proteins that stimulate angiogenesis—can be formulated into topical or injectable products that promote tissue regeneration more effectively. In addition, these fractions can be used to create new biomaterials that integrate the healing properties of latex, providing an ideal support for the repair of damaged tissues. Therefore, a deeper understanding of the biochemical properties of latex and the optimization of relevant separation methods are crucial.

## Figures and Tables

**Table 1:** Protein content of raw latex samples (natural and pre-vulcanized) quantified using elemental analysis and Kjeldahl method.

Sample	Protein Content (g/100 g) Elemental Analysis	Protein Content (g/100 g) Kjeldahl
Natural latex	3.49 ± 0.14	3.05 ± 0.68
Pre-vulcanized latex	3.37 ± 0.11	3.95 ± 0.75

**Table 2:** The nitrogen content of the dialyzed latex serum quantified using different techniques.

Sample	Nitrogen Content (g/100 g) Elemental Analysis	Nitrogen Content (g/100 g) Kjeldahl
Natural latex serum	0.283 ± 0.028	0.998 ± 0.088
Pre-vulcanized latex serum	0.434 ± 0.022	1.070 ± 0.022

**Table 3:** Absorbance in different batches of latex serum obtained in the second Bradford assay.

Sample	Absorbance
Serum Batch 1	0.7734 ± 0.0025
Serum Batch 2 refrigerated dialysate	0.8085 ± 0.0093
Serum Batch 2 frozen dialysate	0.8176 ± 0.0155
Serum Batch 2 non-dialysate	0.9128 ± 0.0040

**Table 4:** Concentrations obtained for the FrHB1, FrHB2, and FrHB3 fractions after the first fractionation experiment.

Sample	Concentration mg/mL
FrHB1 flask 1	0.180 ± 0.002
FrHB1 flask 2	0.135 ± 0.0002
FrHB1 flask 3	0.123 ± 0.002
FrHB2 flask 1	0.130 ± 0.0002
FrHB2 flask 2	0.157 ± 0.007
FrHB2 flask 3	0.123 ± 0.00005
FrHB3 flask 1	0.119 ± 0.0004
FrHB3 flask 2	0.115 ± 0.0006
FrHB3 flask 3	0.114 ± 0.002

**Table 5:** Protein concentrations in the fractions obtained after the second fractionation experiment.

Sample	Concentration (mg/mL)
FrHB1 run 1	0.227 ± 0.002
FrHB2 run 1	0.199 ± 0.002
FrHB1 run 2	0.224 ± 0.001
FrHB2 run 2	0.191 ± 0.006

**Table 6:** Estimated concentration of the samples used for verification of the electrophoretic profile in NuPAGE gel.

Sample	Concentration (mg/mL) *
Soro Lot 2 concentrated	1.994
Soro Lot 2 original	0.319
Soro Lot 2 dilution 1:2	0.995
Soro Lot 2 dilution 1:4	0.49
FrHB1 round 1 concentrated	1.42
FrHB1 round 1 original	0.227
FrHB1 round 1 dilution 1:2	0.114
FrHB1 round 1 dilution 1:4	0.057
FrHB1 round 2 concentrated	1.4
FrHB1 round 2 original	0.224
FrHB1 round 2 dilution 1:2	0.112
FrHB1 round 2 dilution 1:4	0.056

**Table 7:** Protein concentrations and average concentration (in mg/mL) of FrHB1 in the fractions obtained after Fractionation Experiments 5 to 9.

Sample	Concentration (mg/mL)	Average Concentration (mg/mL)
FrHB1 15/03 1	0.231 ± 0.009	
FrHB1 15/03 2	0.233 ± 0.008	
FrHB1 15/03 3	0.208 ± 0.008	
FrHB1 19/03 1	0.202 ± 0.008	
FrHB1 19/03 2	0.214 ± 0.009	
FrHB1 19/03 3	0.219 ± 0.007	
FrHB1 19/03 4	0.216 ± 0.006	
FrHB1 19/03 5	0.205 ± 0.001	
FrHB1 20/03 1	0.211 ± 0.016	0.196 ± 0.030
FrHB1 20/03 2	0.197 ± 0.003	
FrHB1 20/03 3	0.182 ± 0.002	
FrHB1 26/03 1	0.192 ± 0.013	
FrHB1 26/03 2	0.163 ± 0.001	
FrHB1 26/03 3	0.161 ± 0.002	
FrHB1 01/04 1	0.155 ± 0.007	
FrHB1 01/04 2	0.142 ± 0.001	
FrHB1 01/04 3	0.131 ± 0.001	

## Data Availability

All relevant information is contained within the article. However, if further details are required, please feel free to contact the corresponding author.
